# Patient-reported measures outcomes: modern evaluation of oral health

**DOI:** 10.1186/s12903-023-03219-0

**Published:** 2023-07-18

**Authors:** Dhelfeson Willya Douglas-de-Oliveira, Kitty Jieyi Chen

**Affiliations:** 1 Department of Dentistry, Federal University of Jequitinhonha and Mucuri Valleys, Rua da Glória, 187, Centro, Diamantina, Minas Gerais 39100-000 Brazil; 2 Hospital of Stomatology, Guanghua School of Stomatology, Guangdong Provincial Key Laboratory of Stomatology, Sun Yat-sen University, No. 56 Lingyuan West Road, Yuexiu District, Guangzhou City, Guangdong Province 510080 China

**Keywords:** Patient-reported outcomes, Quality of life, Oral health, Editorial, Patient-centered care

## Abstract

Patient reported outcomes have become important, with instruments (patient reported outcomes measures - PROMs) being used to assess treatment success and patient satisfaction. In this editorial, the dental PROM context is presented, and submissions are invited for a special collection from *BMC Oral Health* entitled ‘Patient-reported outcomes in dentistry’.

Historically, clinical dental research primarily relied on clinical inputs from dental professionals. Outcome assessment of dental treatments and oral care emerged on how long they last, whether failure or complications occur, healing rate, survival time assessment, long-term success, identification of risk and protective factors, effects of new materials and techniques, and evaluation of clinical parameters. These outcomes represent the clinician-observed impact of the oral condition or the dental intervention and are more or less objectively measurable [1]. They are used due to the assumption that these outcomes are related to biological and technical matters.

However, there were concerns that clinical outcomes do not adequately reflect the patients’ experience. Consequently, patient opinion and satisfaction are increasingly becoming important in healthcare research [2]. A patient-reported outcome (PRO) is defined as any report of the status of a patient’s health condition that comes directly from the patient, without interpretation of the patient’s response by a clinician or anyone else, describing how they feel about a condition or therapy [3]. Patient reporting helps to improve doctor-patient communication, patients’ satisfaction and symptom management.To assess a PRO, a patient-reported outcome measure (PROM), most often a self-administrated questionnaire, is used.

In the dental field, PROMs have become essential in evaluating dental treatments’ success. One of the first socio-dental indicators on people`s well-being was the Social Impacts of Dental Disease (SIDD) scale [4]. The SIDD scale aimed to measure patients’ oral health and quality of life by assessing five categories of impact: eating restrictions, communication restrictions, pain, discomfort and aesthetic dissatisfaction. Since then, numerous questionnaires have been developed to measure treatment outcomes and used successfully in different dental specialties. As example, we can cite the Geriatric Oral Health and Assessment Index (GOHAI), Dental Impact Profile (DIP), Oral Health Impact Profile (OHIP), Craniofacial Pain and Disability Inventory (CF-PDI) and Dentine Hypersensitivity Experience Questionnaire (DHEQ) [5]. The development, validation, and use of PROMs in Dentistry have revolutionized the approach to assess treatment outcomes and provide insights into how treatments can be improved to better reflect patients’ experiences, health behaviors and needs.

The most widely used PROMs are questionnaires having multiple questions in order to capture specific or general oral conditions or diseases for use with younger and older populations [2]. However, there are alternative tools that can be used to estimate the patient’s satisfaction and health status at a particular point in time. For example, numerical rating scales, verbal rating scales, face scales, and visual analogue scales are easy instruments frequently used to capture the patient’s subjectivity related to well-being, mainly pain [6]. In general, these questionnaires and scales are processed within a methodology to provide a metric.

PROMs are relevant to all dental specialties, as they provide valuable information on how treatments affect patients’ physical, psychological, and social well-being. Measuring PROs allows for a patient-centered approach to dental care, where treatment success is defined by the patient’s perspective. Current research highlights the importance of using validated instruments and questionnaires [3, 7].

PROMs can provide critical information to clinicians, researchers and policymakers, indicating how well a particular treatment works and how it can be improved considering the patient’s or caregiver’s point of view. Besides, professional training for dental students in most dental schools focus on clinical outcomes. But outcome assessment should be patient-centered and patient-oriented as required by personalized medicine to improve health outcomes for individual patients. More evidence of PROs in dentistry are needed for dental educators before integrating PROs into dental education.

It is essential for dental practitioners, researchers and educators to consider PROs in treatment planning, professional training and decision-making to provide more patient-centered care. This perspective introduces the *BMC Oral Health* Collection on Patient-reported outcomes in dentistry. This special issue considers the patient as the center of attention for dental treatment success; it also focuses on patients’ perspectives and measures about dental care and the effect of treatments on their oral health and quality of life.

## Data Availability

Not applicable.

## List of abbreviations

PRO: Patient-reported outcome
PROM: Patient-reported outcome measure
SIDD: Social Impacts of Dental Disease
GOHAI: Geriatric Oral Health and Assessment Index
DIP: Dental Impact Profile
OHIP: Oral Health Impact Profile
CF-PDI: Craniofacial Pain and Disability Inventory
DHEQ: Dentine Hypersensitivity Experience Questionnaire
